# Evaluation of dihydrofolate reductase and dihydropteroate synthetase genotypes that confer resistance to sulphadoxine-pyrimethamine in *Plasmodium falciparum* in Haiti

**DOI:** 10.1186/1475-2875-11-275

**Published:** 2012-08-13

**Authors:** Tamar E Carter, Megan Warner, Connie J Mulligan, Alexander Existe, Yves S Victor, Gladys Memnon, Jacques Boncy, Roland Oscar, Mark M Fukuda, Bernard A Okech

**Affiliations:** 1 Genetics Institute, University of Florida, 2033 Mowry Road, PO Box 103610, Gainesville, FL 32610, USA; 2 Department of Anthropology, University of Florida, Turlington Hall, Room 1112, PO Box 117305, Gainesville, FL 32611, USA; 3 Department of Epidemiology, College of Public Health and Health Professions, University of Florida, 1225 Center Drive, Room 3101, PO Box 100231, Gainesville, FL 32611, USA; 4 Emerging Pathogens Institute, University of Florida, 2055 Mowry Rd, P.O. Box 100009, Gainesville, FL 32610, USA; 5 National Public Health Laboratory, Ministry of Public Health and Population (MSPP), Port au Prince, Haiti; 6 Blanchard Clinic, Family Health Ministries Haiti, Terre Noire, Port au Prince, Haiti; 7 Hospital Saint Croix, Leogane, Haiti; 8 National Malaria Control Program, Ministry of Public Health and Population, Port au Prince, Haiti; 9 Armed Forces Health Sciences Surveillance Center, 11800 Tech Road, Suite 220, Silver Spring, MD 20904, USA; 10 Department of Environmental and Global Health, University of Florida, PO Box 100188, Gainesville, FL 32610, USA

**Keywords:** Malaria, Hispaniola, Folic acid antagonists, Anti-malarials, Drug resistance, Transmission, Fansidar

## Abstract

**Background:**

Malaria caused by *Plasmodium falciparum* infects roughly 30,000 individuals in Haiti each year. Haiti has used chloroquine (CQ) as a first-line treatment for malaria for many years and as a result there are concerns that malaria parasites may develop resistance to CQ over time. Therefore it is important to prepare for alternative malaria treatment options should CQ resistance develop. In many other malaria-endemic regions, antifolates, particularly pyrimethamine (PYR) and sulphadoxine (SDX) treatment combination (SP), have been used as an alternative when CQ resistance has developed. This study evaluated mutations in the dihydrofolate reductase (*dhfr*) and dihydropteroate synthetase (*dhps*) genes that confer PYR and SDX resistance, respectively, in *P. falciparum* to provide baseline data in Haiti. This study is the first comprehensive study to examine PYR and SDX resistance genotypes in *P. falciparum* in Haiti.

**Methods:**

DNA was extracted from dried blood spots and genotyped for PYR and SDX resistance mutations in *P. falciparum* using PCR and DNA sequencing methods. Sixty-one samples were genotyped for PYR resistance in codons 51, 59, 108 and 164 of the *dhfr* gene and 58 samples were genotyped for SDX resistance codons 436, 437, 540 of the *dhps* gene in *P. falciparum*.

**Results:**

Thirty-three percent (20/61) of the samples carried a mutation at codon 108 (S108N) of the *dhfr* gene. No mutations in *dhfr* at codons 51, 59, 164 were observed in any of the samples. In addition, no mutations were observed in *dhps* at the three codons (436, 437, 540) examined. No significant difference was observed between samples collected in urban *vs* rural sites (Welch’s T-test p-value = 0.53 and permutations p-value = 0.59).

**Conclusion:**

This study has shown the presence of the S108N mutation in *P. falciparum* that confers low-level PYR resistance in Haiti. However, the absence of SDX resistance mutations suggests that SP resistance may not be present in Haiti. These results have important implications for ongoing discussions on alternative malaria treatment options in Haiti.

## Background

Malaria causes almost half a million deaths worldwide every year, with *Plasmodium falciparum* accounting for most of the deaths [1]. In Haiti, roughly 30,000 people contract malaria annually [2], making it a significant public health concern for the country. The treatment for malaria in Haiti has relied on chloroquine (CQ) for several decades [3,4]. However, there is evidence that CQ-resistance genotypes may be emerging in Haiti [5,6] and there are ongoing discussions about the need to incorporate alternative treatments for the management of malaria.

Many malaria endemic countries that reported CQ resistance switched to antifolate treatments, especially the combination treatment of pyrimethamine (PYR) and sulphadoxine (SDX) [7]. In Haiti, PYR was first introduced in the early 1960s during the global effort to eliminate malaria [3]. The Haiti Ministry of Health, in partnership with the World Health Organization, worked to reduce malaria in Haiti by incorporating PYR in addition to CQ which was already being used [3] and insecticide spraying to kill mosquitoes. After a decade of use, PYR-resistant strains of *P. falciparum* were reported in Haiti based on *in vitro* studies [8], but no SP resistance was observed *in vivo*[8]. Since 1985, there have been no additional comprehensive studies to examine SP resistance in Haiti [9].

The *in vitro* and *in vivo* resistance of *P. falciparum* to PYR and SDX has been associated with single point mutations in the dihydrofolate reductase (*dhfr*) [10-12] and dihydropteroate synthase (*dhps*) genes [13-15], respectively. These same point mutations have also been associated with SP resistances. Correlations have been found between SP resistance and point mutations at *dhfr* codons 51, 59, 108, and 164 and *dhps* codons 436, 437, and 540 [16-20]. The mutation at codon 108 in *dhfr* is the first to develop in a population under pressure from PYR use [21,22]. The stepwise evolution of additional mutations, particularly at codons 51 and 59, directly correlates with increased resistance to PYR [10,12,23,24]. To date, no comprehensive molecular studies on *dhfr* and *dhps* genotypes associated with resistance to PYR and SDX, respectively have been conducted in Haiti beyond a single study that examined only three samples [25].

In this study, genetic markers for SP resistance in the *dhfr* and *dhps* genes were assayed in *P. falciparum* samples from Haiti. The data obtained from this study are important for future anti-malaria drug health policy discussions for Haiti and for understanding the evolution of drug resistance in *P. falciparum*.

## Methods

### Study sites

**Figure 1:**
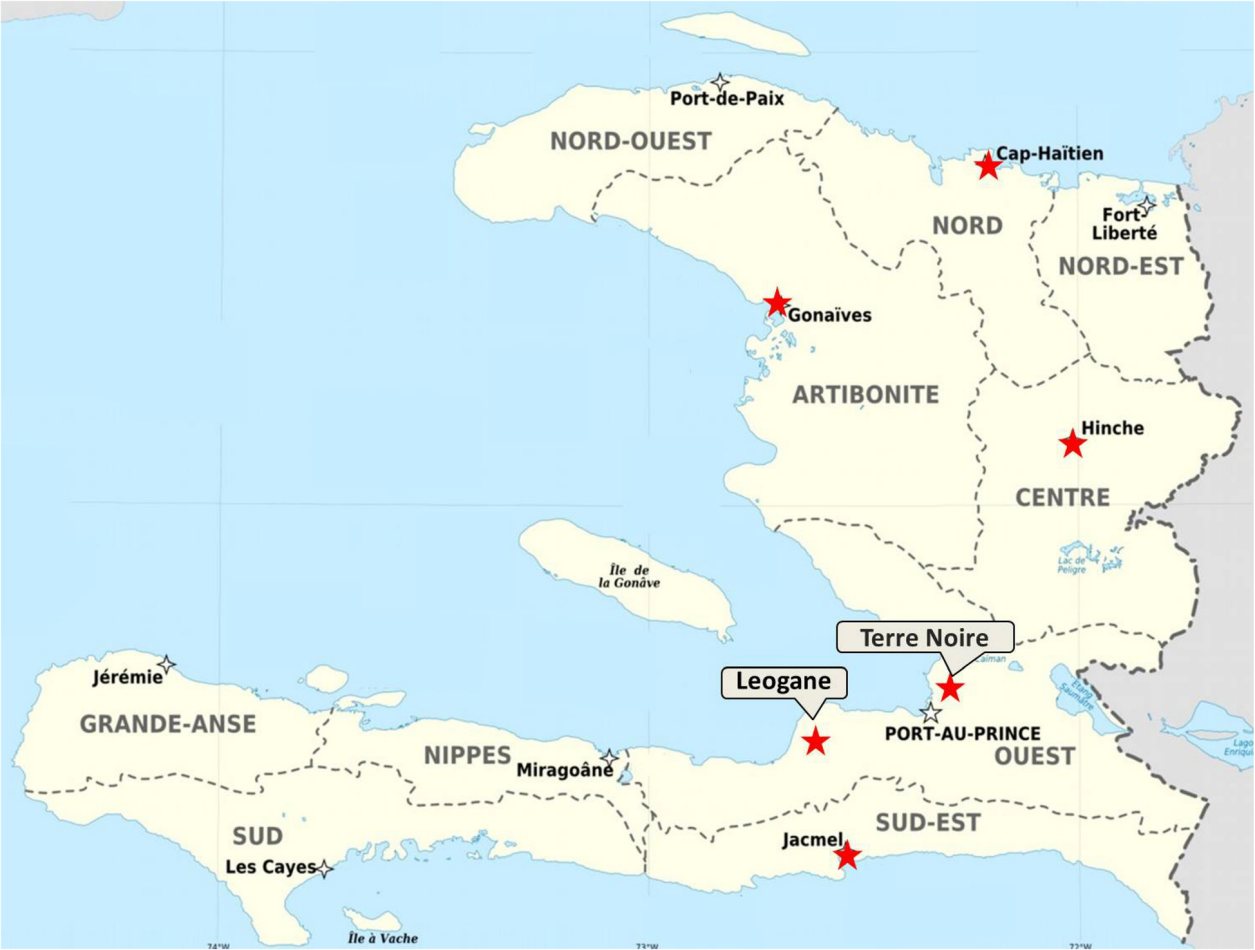
Map of sample collection sites (red stars) in Haiti.

The blood samples were collected from six sites: Terre Noire in Port au Prince at the Blanchard Clinic (TN) and Leogane at the Hospital Sainte Croix (LN), Artibonite (ART), Hinche (HN), Cap Haitien (CP), and Jacmel (JM) (Figure 1). The TN community is very close to Port au Prince international airport and, therefore, is categorized as urban although the public health infrastructure is very poor. LN, ART, HN, CP, and JM are located in rural communities. The rural communities primarily engage in subsistence farming and informal trade for income. There is very limited electricity and piped water. Spring water that feeds into seasonal rivers is a part of the landscape and a source for domestic water that contributes to the growth of malaria-transmitting mosquitoes. There is no active malaria control activity in the area.

### Sample collection

Dried blood spots samples were obtained from 319 individuals. The samples were collected in Haiti between May 2010 and February 2012. Table 1 includes a summary of the sample collection from each site. Two separate inclusion criteria were used for sample collection. The first 196 samples were selected based on presentation of malaria-like symptoms (Sample Set 1) and were collected only from TN. The other 123 samples (Sample Set 2) were included based on positive *P. falciparum* detection using microscopy and included samples from TN (n = 36), LN (n = 31), ART (n = 25), HN (n = 10), CP (n = 10), and JM (n = 11). All malaria-infected patients were treated according to the standard policy available at the clinics. The study was approved by the Haiti Ethical Review Board, UF-IRB, and the Office of Research Protections, US Army Medical Research and Materiel Command (USAMRMC).

**Table 1:** Sampling summary

**Study Site**	**Classification**	**No. of samples tested**	**No. presumptively diagnosed based on symptoms**	**No. confirmed by microscopy**
Terre Noire	Urban	232	196	36
Leogane	Rural	31	0	31
Artibonite	Rural	25	0	25
Hinche	Rural	10	0	10
Cap Haitien	Rural	10	0	10
Jacmel	Rural	11	0	11
Total	--	319	0	123

### DNA extraction

DNA was extracted from dried blood spots using a methanol wash protocol as previously described [26]. Two 1.2 mm punches were taken from each dried blood spot sample and placed in 0.2 ml tubes. The punches were soaked in methanol at room temperature for 15 min. The methanol was then removed and the punches air-dried for about 30 min. Sterile DNA grade water (65 ul) was then added to each tube and the tubes were heated to 97°C for 15 min.

### PCR amplification

To detect the presence of *P. falciparum* DNA in the samples, the small subunit ribosomal RNA (*ssu rRNA*) gene of *P. falciparum* was amplified as described in Londono *et al.*[5]. Subsequent amplification and genotyping of *dhfr* and *dhps* was conducted on all Set 1 samples with positive *ssu rRNA* results and all Set 2 samples. Regions of the *dhfr* (522 bp) and *dhps* (438 bp) genes were amplified using a nested PCR protocol as previously described [27] except that the amplicons were sequenced instead of assayed by restriction digestion. Primer sequences for each nested step and the nucleotide location are listed in Table 2. All PCR reactions included reagents at the following concentrations: 1X GoTaq Flexi Buffer, 0.75 mM MgCl_2_, 0.2 mM each nucleotide, 0.25 μM for each primer, and 1.25 U of Go Taq Hot Start Polymerase (Promega, Madison, WI). The temperature protocol used for all primary PCRs (PCR 1) was as follows: initial denaturation at 94°C for 3 min, 40 cycles of denaturation at 94°C for 1 min, annealing at 45°C for 2 min, extension at 72°C for 2 min, and a final extension at 72°C for 10 min. The temperature protocol used for the nested PCRs (PCR 2) was as follows: initial denaturation at 94°C for 3 min, 40 cycles of denaturation at 94°C for 1 min, annealing at 50°C for 2 min, extension at 72°C for 2 min, and a final extension for 10 min.

**Table 2:** Primer sequences used for nested PCR protocol and sequencing

**Gene**	**Primer**	**Sequence**^**‡**^	**Nucleotide location**	
*dhfr*	PCR1	M1*	TTTATGATGGAACAAGTCTGC	−3 to 18
		M5*	AGTATATACATCGCTAACAGA	625 to 645
	PCR2	M3*	TTTATGATGGAACAAGTCTGCGACGTT	−3 to 24
		F/*	AAATTCTTGATAAACAACGGAACCTTTTA	491 to 519
*dhps*	PCR1	R2*	AACCTAAACGTGCTGTTCAA	223 to 242
		R/*	AATTGTGTGATTTGTCCACAA	913 to 933
	PCR2	K*	TGCTAGTGTTATAGATATAGGATGAGCATC	269 to 298
		K/*	CTATAACGAGGTATTGCATTTAATGCAAGAA	676 to 706
	Seq	Kb^†^	ATTGGTTTCGCATCACATTT	408 to 427

‡Primer sequences listed as forward primer first followed by reverse primer. All primers listed 5’ to 3’.

*Primers taken from Duraisingh *et al.*[27].

†Primer designed for nested sequencing of *dhps* codon 436 and 437.

### Sequencing and alignment

PCR products were Sanger sequenced at the University of Florida’s Interdisciplinary Center for Biotechnology Research DNA Sequencing Core Laboratory with BigDye^TM^ chemistry (Applied Biosystems, Foster City, CA) on an Applied Biosystems 3730 Genetic Analyzer. The PCR 2 forward and reverse primers were used for sequencing the *dhfr* and *dhps* PCR amplicons. An additional primer (Kb, see Table 2 for primer sequence) was designed for nested sequencing of *dhps* amplicons that gave poor sequences with the PCR 2 primers for the region including codons 436 and 437. Sequence data were aligned to *P. falciparum* 3D7 sequences [GenBank: XM_001351443 and XM_001349382] using *Sequencher 4.10.1*. (Gene Codes Corp). The *dhfr* and *dhps* gene sequences were scanned for previously identified *dhfr* and *dhps* point mutations in resistance-associated codons (see Table 3).

**Table 3:** List of wild type and resistance codon sequences

**Gene**	**Codon**	**Wild type codon(s) (amino acid)**	**Resistance codon(s) (amino acid)**
*dhfr*	51	AAT, AAC (N)	ATT (I)
	59	TGT (C)	CGT (R)
	108	AGC (S)	AAC (N), ACC (T)
	164	ATA(I)	TTA (L)
*dhps*	436	TCT (S)	GCT (A), TTT (F)
	437	GCT (A)	GGT (G)
	540	AAA (K)	GAA (E)

### Statistical analysis

Welch’s t-test and permutations were used to determine if there was a significant difference in the number of samples with detectable levels of *P. falciparum* DNA (ie, positive PCR results with *ssu rRNA, dfhr*, or *dhps*) between Sample Set 1 and Sample Set 2. Additionally, the proportion of samples that carried each resistance-associated mutation was determined. Welch’s t-test and permutations were used to compare the proportion of samples with mutations between samples collected in an urban region (TN) to samples collected in a rural region (LN, CP, HN, and JM). All statistical analysis were completed using the open source software package R version 2.14.1 [28].

## Results

### Sampling summary and positive amplification results

A total of 319 samples were collected in this study. Sample Set 1 included 196 samples and Sample Set 2 included 123 samples (see Table 1). A summary of amplification results for the three genes is presented in Table 4. The proportion of samples with detectable levels of *P. falciparum* DNA (i e, positive amplification of *ssu rRNA*, *dhfr*, or *dhps*) was compared between Sample Set 1 and Sample Set 2 for the TN site. The proportions (Sample Set 1 = 0.07 and Sample Set 2 = 0.72) were determined to be significantly different as assessed by Welch’s T-test and permutation (p-value < < 0.001).

**Table 4:** Amplification and sequencing results

**Study Site**	**Total samples**	**# Positive amplifications**			**# Sequenced**		
		** *ssu rRNA* **	** *dhfr* **	** *dhps* **	** *dhfr* **	** *dhps* **	** *Both dhfr and dhps* **
Terre Noire †	232	39 (SS1 =13, SS2 =26)	33 (SS1 =12, SS2 =21)	31 (SS1 = 10, SS2 = 21)	30 (SS1 = 12, SS2 = 18)	23 (SS1 = 10, SS2 = 13)	21 (SS1 = 10, SS2 = 11)
Leogane	31	24	14	18	13	13	10
Hinche	10	6	5	8	5	8	4
Cap Haitien	10	1	4	8	4	7	4
Jacmel	11	6	9	8	9	7	7
Total*	294	76	65	73	61	58	46

†Includes the number of samples for samples set (SS) in parentheses; SS1 = Sample Set 1 and SS2 = Sample Set 2.

*Total does not include Artibonite samples. None of the Artibonite samples contained positive amplifications for *ssu rRNA*, *dhfr*, or *dhps*.

The *dhfr* and *dhps* genes were amplified in Set 1 samples with positive *ssu rRNA* results and Set 2 samples. Of these samples, 65 were successfully PCR amplified for the *dhfr* gene and 61 samples were successfully sequenced [Genbank: JX217825 – JX217828]. Likewise, the *dhps* gene was successfully amplified in 73 samples and 58 samples were successfully sequenced. Finally, complete and matched *dhfr* and *dhps* sequence data were available for 46 samples.

### *Dhfr* and *dhps* mutations

Of the 61 samples that were successfully sequenced for *dhfr,* 20 samples (32.79%) carried the S108N mutation (Table 5) [Genbank: JX217825]. Wild type alleles were observed in all samples at *dhfr* codons 51, 59, and 164 (Table 5). Other studies have reported two possible wild type alleles for the *dhfr* codon 51, AAT and AAC, both coding for asparagine, (N) (Table 3), but only the AAT allele was observed in this study’s samples. In addition to the S108N mutation, a mutation was observed in codon 62 in a single sample that resulted in an amino acid change from threonine, (T) to leucine, (L) [Genbank: JX217827]. Another mutation was observed in codon 158 of two samples that resulted in an amino change from aspartic acid to glycine [Genbank: JX217825]. No mutations were observed in the *dhps* gene in the samples.

**Table 5:** Percent of samples with S108N mutation for each collection site

**Study Site**	**# DHFR sequenced**	**S108N mutants (%)**
Terre Noire	30	11 (36.67%)
Leogane	13	2 (13.33%)
Hinche	5	0 (0%)
Cap Haitien	4	1 (25%)
Jacmel	9	6 (66.67%)
Total	61	20 (32.79%)

### Urban and rural S108N frequency comparison

**Figure 2:**
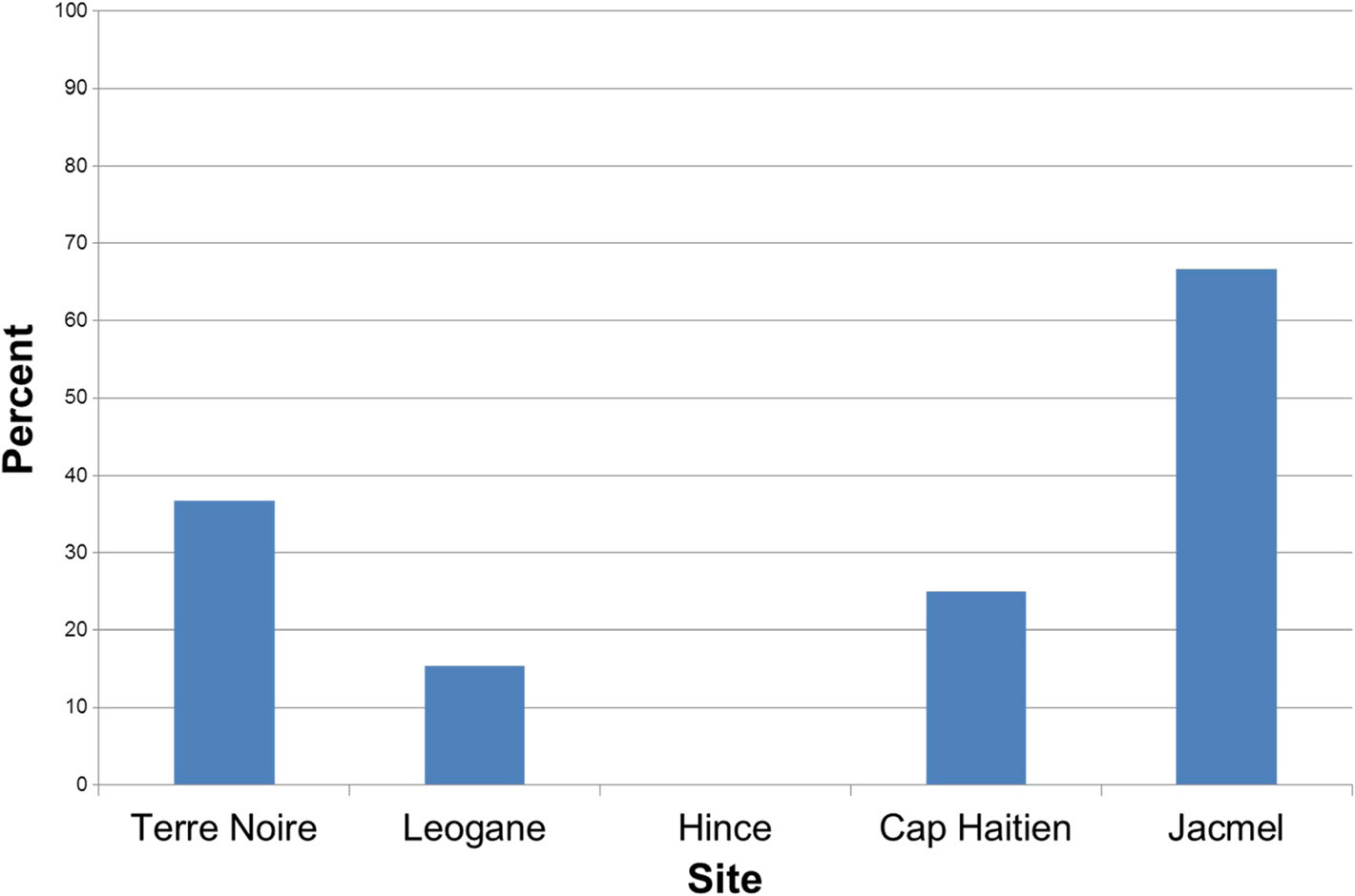
Comparison of the prevalence of S108N mutation by collection site.

Of the 61 samples that were successfully sequenced for *dhfr,* 30 samples were collected from an urban site (TN) and 31 were collected from rural sites (LN, HN, CP, and JM). The proportion of samples with the S108N mutation in each study site is represented in Figure2. Eleven (36.67%) of the samples collected from the urban site and nine (29.03%) from the rural sites had the S108N mutation. The proportion of samples with the S108N mutation did not differ significantly between the samples collected in urban *vs* rural sites, based on Welch’s T-test (p-value = 0.53) and permutations (p-value = 0.59), suggesting that that samples collected from the urban site are not significantly more likely to carry the S108N mutation than the samples from the rural site.

## Discussion

### Sampling summary and presence of *Plasmodium falciparum* DNA

The purpose of the present study was to investigate the presence of SP resistance associated mutations in the *dhfr* and *dhps* genes in *P. falciparum* in Haiti. As reported in Table 1, a total of 319 samples were collected. However, only 76 samples, or 24%, had positive *ssu rRNA* amplification. An additional 16 samples from Set 2 amplified *dhfr* or *dhps*, although they did not successfully amplify *ssu rRNA*. Furthermore, there was a significant disparity in the number of samples with detectable levels of *P. falciparum* DNA (i e, positive amplification for *ssu rRNA, dhfr*, or *dhps*) between the samples that were collected based on malaria-like symptoms (Sample Set 1) and microscopy (Sample Set 2) from TN (p-value < <0.001). It is likely that malaria is often over-diagnosed in Haiti as the symptoms of malaria (fever, chills, fatigue, etc.) are similar to that of other diseases, thus the samples from Set 1 were less likely to contain *P. falciparum* DNA.

### PYR-resistance mutations

Sixty-one samples were sequenced successfully for *dhfr* and 20 of these samples carried the S108N *dhfr* mutation associated with PYR resistance. No other resistance mutations genotyped in the *dhfr* gene were observed. Previous studies have reported that the S108N mutation is essential to PYR resistance [10,11,29] and is the first mutation seen as PYR resistance develops [21,22]. The single S108N mutation is associated with a lower level of resistance to PYR relative to multi-allelic resistance associated with S108N plus other mutations at codon positions 51, 59, and 164 [10,12,23,24]. Therefore, these results suggest that there may be PYR resistance in Haiti and that the resistance would be low based on the absence of multiple mutations at codons 51, 59, and 164 in the *P. falciparum* samples analysed.

Continued presence of PYR-resistant S108N-only haplotypes in Haiti decades after PYR use was discontinued seems surprising. In this study, only the single S108N haplotype was observed, while in other studies the S108N mutation is rarely observed alone. Recent studies of discontinued use of SP in Peru, followed up to five years, have noted a decrease in multiple mutation *dhfr* haplotypes while noting an increase in S108N only haplotypes [30,31]. This may be due to a greater fitness cost for multiple mutation haplotypes in the absence of antifolate drug pressure [31]. However, no studies have documented a complete loss of S108N haplotypes following discontinued use of SP; thus, it is not possible to calculate how quickly S108N may be lost. Furthermore, studies have not found evidence of strong selective pressure on the S108N-only haplotype [32] suggesting that there may be minimal fitness costs associated with maintaining only the S108N allele. Another possible explanation for the presence of a PYR-resistant mutation in Haiti is that privately run clinics funded by charities and unregulated donations of medications may be using SP regimes for the clinical management of malaria in Haiti. There is no evidence that this is happening but the lack of government oversight of private clinics run by charities may lead to unauthorized medications being used. There is also the possibility of cross-resistance due to sulphonamide-based treatments for HIV that act on the folate pathway and may induce mutations in *P. falciparum dhfr* gene [33]. Iyer *et al.* found that *P. falciparum* strains with the S108N mutation were resistant to the sulphadmide-based HIV treatment trimethoprim [33], suggesting that widespread use of trimethoprim could result in selection of resistance mutations in the *dhfr*. Another possibility is that the S108N mutation may have been introduced from South American countries, such as Bolivia, Columbia, or Peru where PYR or SP has been used or is being used. For example, a study in Peru reported a 79% prevalence of the single S108N haplotype [31]. Corredor *et al.* also reported an increase in the single S108N haplotypes to 26% in the Amazon basin in Colombia [34]. Few studies have examined the presence of *dhfr* resistance haplotypes in Central America, with the exception of Jovel *et al.*[35] (Honduras) and Samudio *et al.*[36] (Panama). Neither of these studies observed the single S108N haplotype in their samples. Investigations into the origin of the *dhfr* S108N mutation in Haiti could provide insight into how drug resistance mutations arise and spread throughout a population.

Two additional mutations in the *dhfr* (T62L and D148G) that have not previously been reported were observed in this study. Both mutations result in a change in side-chain polarity of the amino acid (polar to non-polar) and the D148G mutation results in a change of side-chain charge, suggesting that these mutations may affect *P. falciparum* functionality. Further studies are needed to investigate whether these mutations affect resistance to PYR.

### SDX resistance mutations

The lack of mutations in the *dhps* genes in the samples suggests that no drug pressure has been acting upon the gene. Further studies are recommended to increase the sample size and sampling sites and to conduct *in vitro* sensitivity studies on SDX in malaria parasites from Haiti. Nonetheless, based on the data in this paper, the lack of mutations in the *dhps* may indicate that *P. falciparum* parasites in Haiti are still sensitive to SDX.

## Conclusion

The finding of the *dhfr* S108N mutation would suggest that PYR-resistant *P. falciparum* may still be present in Haiti, at least at a low level. However, S108N alone has not been associated with SP resistance and, thus, the use of combination SP for the treatment of malaria in Haiti should be considered as a replacement medication in the event that CQ resistance emerges.

## Competing interests

The authors declare that they have no competing interests.

## Authors’ contributions

TEC contributed to the design of the study, completed molecular genetic studies, statistical analysis and interpretation, and drafted the manuscript. MW generated a portion of the molecular data. CJM contributed to the study design, interpretation of results, and the writing of the manuscript. AE organized collection of samples in Haiti and interpretation of data. JSV, GM, JB and RO collected samples in Haiti. MF assisted with the drafting of the manuscript. BAO designed the study, facilitated sample collection in Haiti, and contributed interpretation of the data and the drafting of manuscript. All authors read and approved the final manuscript.
